# Control and prevention strategies of porcine respiratory disease complex: current evidence and future directions

**DOI:** 10.3389/fvets.2026.1992010

**Published:** 2026-10-27

**Authors:** Ana Isabel Pastor-Calonge, Óscar Mencía-Ares, Lucía Manzanares-Vigo, Alejandro Cid-González, César Bernardo Gutiérrez-Martín, Sonia Martínez-Martínez

**Affiliations:** 1 Department of Animal Health, Faculty of Veterinary, Universidad de León, León, Spain; 2 Instituto de Desarrollo Ganadero y Sanidad Animal (INDEGSAL), Universidad de León, León, Spain

**Keywords:** antimicrobial alternatives, biosecurity, coinfections, essential oils, probiotics, respiratory pathogens, swine, vaccines

## Abstract

Porcine Respiratory Disease Complex (PRDC) is a multifactorial disease with a major economic impact on the global swine industry. Its emergence and progression are strongly influenced by interactions among infectious agents, environmental conditions, and management practices, making its prevention and control particularly challenging. This review summarizes the principal strategies and emerging approaches for PRDC prevention and control, highlights recent advances, and discusses their potential limitations and future research needs. Effective PRDC management requires a comprehensive and multifaceted approach that accounts for the high frequency of coinfections involving multiple pathogens and the overlapping and nonspecific clinical signs associated with respiratory disease. Accordingly, control programs should integrate external and internal biosecurity measures. For external biosecurity, the priority is to prevent pathogen introduction through appropriate animal sourcing, quarantine and acclimation procedures, semen screening, pest control, and management of transport, personnel, and fomites. Within farms, internal biosecurity measures should then limit pathogen transmission through appropriate pig flow and herd management, stocking density control, and optimization of environmental conditions. Because pathogen detection alone does not establish its role in disease, diagnosis should combine clinical, epidemiological, pathological, and laboratory findings. Continued herd-level monitoring, supported by appropriate sampling and laboratory testing, is also essential for identifying changes in pathogen circulation and evaluating control measures. Vaccination remains a cornerstone of PRDC prevention, while emerging vaccine technologies and novel immunization strategies offer promising opportunities to improve protection against respiratory pathogens. Furthermore, growing concerns regarding antimicrobial resistance have intensified the search for alternatives to conventional antibiotics, including probiotics, essential oils, and bacteriophages, which have shown encouraging potential but require further validation under field conditions. Overall, successful PRDC prevention and control depend on recognizing its multifactorial nature and integrating biosecurity, vaccination, management practices, antimicrobial alternatives, and effective monitoring systems. Further research conducted under commercial production conditions will be essential to determine the practical efficacy of emerging approaches and facilitate their incorporation into comprehensive and sustainable PRDC control programs.

## Introduction

Porcine respiratory disease complex (PRDC) is considered a multifactorial set of diseases that greatly affects the porcine sector worldwide. This disease complex represents a significant economic concern for the swine industry, with high morbidity and mortality rates (1). In addition, it often progresses to a chronic state, aggravating long-term production losses. The financial losses have been estimated to amount to 2.30–15.35 € per fattening pig per year or 100–323 € per sow per year (2). Elements such as environmental stress, production systems, host susceptibility, and farm management practices can all contribute to conditions that favor the development and spread of the pathogens involved, ultimately having a major negative impact on pig health (3).

PRDC is represented by a set of diseases caused by an array of different pathogens and characterized, especially in the early stages, by a nonspecific clinical presentation like coughing, fever or lethargy. Primary pathogens are the ones that facilitate the establishment of secondary infections by damaging the respiratory tract or impairing the immune system. These include *Mesomycoplasma hyopneumoniae*, *Actinobacillus pleuropneumoniae*, porcine reproductive and respiratory syndrome virus (PRRSV), porcine circovirus type 2 (PCV2), swine influenza A virus (swIAV). Pseudorabies virus (PRV) and porcine respiratory coronavirus (PRCoV) have historically been considered as primary respiratory pathogens that can participate in PRDC. However, in modern commercial pig production they are generally regarded as minor or infrequent contributors (4). In the case of PRV, this was largely achieved through successful control and eradication programmes in many regions (5).

Secondary pathogens, which normally exist as part of the respiratory tract’s commensal microbiota, can proliferate following infection by primary pathogens. Notable examples of such opportunistic agents include *Pasteurella multocida*, *Glaesserella parasuis*, *Streptococcus suis*, and porcine cytomegalovirus (PCMV). However, the binary classification of primary versus secondary pathogens should be regarded as a useful framework rather than a strict distinction, as the role of some bacterial pathogens may vary depending on factors such as host age and infection context. For instance, *Bordetella bronchiseptica* can act as a primary pathogen in young piglets, whereas it may contribute as a secondary pathogen in older pigs (4).

Parasites, such as the nematodes *Metastrongylus* spp. and *Ascaris suum*, may also contribute to respiratory disease; however, their role is generally considered minor especially in modern intensive production systems (6, 7).

The multifactorial and polymicrobial nature of this complex, in addition to the high coinfection rates often detected and the nonspecific respiratory signs, demands holistic and multifaceted prevention and control approaches (8). These approaches should include strengthened biosecurity, revised management practices, and appropriate medical interventions, especially vaccination, which adds up to the economic burden of this disease (2).

Major challenges in the assessment and control of PRDC still remain in modern pig production. The wide diversity of pathogens involved, together with frequent coinfections that produce overlapping clinical signs, complicates accurate diagnosis and hinders the implementation of targeted preventive measures. Therefore, understanding pathogen interactions and coinfections is essential to inform preventive and therapeutic strategies (9). These difficulties are further exacerbated by the ability of many PRDC-associated pathogens to persist in the environment or within chronically infected carrier animals, often facilitated by biofilm formation (10). In addition, the rapid mutation rates observed in certain microorganisms substantially limit the effectiveness of control efforts (11, 12). Therefore, the objective of this review is to analyze the most effective and recent prophylactic strategies used to control PRDC, to assess their potential limitations, and to discuss the future perspectives and emerging directions in this field.

## Bibliography search

Bibliography selection was based on the results obtained from Google Scholar and PubMed using the search terms “porcine respiratory disease complex”, “control” and/or “prevention” and “pig” or “swine.” Additional searches were performed by replacing “porcine respiratory disease complex” with the specific names of PRDC-related pathogens; and “control” and “prevention” with terms referring to specific preventive or control strategies (e.g., “vaccines,” “probiotics,” “management,” etc). Literature was searched during March 2025– September 2026. Results were initially screened according to relevance, first restricting the search to publications from the previous 5 years to capture the most recent developments, and subsequently without a defined publication period to identify relevant earlier studies. Research articles, review articles, and book chapters from reputable scientific and academic sources, as well as reports from recognized research institutions and relevant official or regulatory documents, were considered suitable sources. Search results were initially screened based on their titles. Abstracts were screened for relevant concepts on specific strategies or approaches of interest (e.g., “virus-like particles” “mRNA vaccine,” “nano-encapsulated essential oil”) and the full texts of potentially relevant articles were subsequently reviewed.

## The value of strong biosecurity

External and internal biosecurity measures are essential and complementary components in the control of PRDC. While external biosecurity focuses on preventing pathogen introduction, internal biosecurity limits their spread and persistence within the herd once present. The consistent and rigorous application of both strategies is therefore critical to reduce infection pressure, improve herd health, and enhance the overall effectiveness of additional interventions such as vaccination or medical treatments (Figure 1).

**Figure 1:**
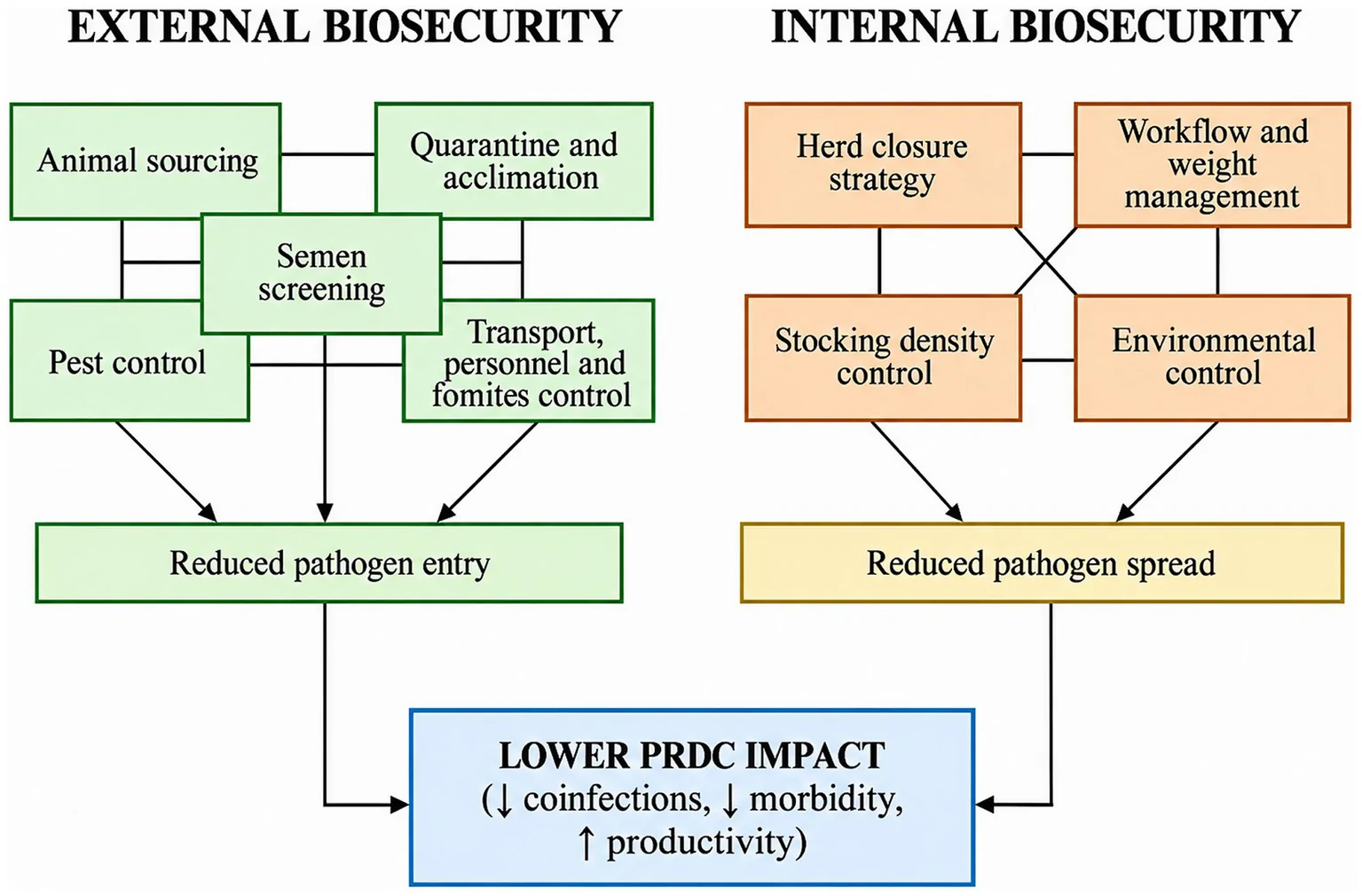
Diagram of integrated biosecurity in the management of PRDC. External biosecurity measures aim to prevent pathogen introduction into the herd, while internal biosecurity measures seek to limit pathogen spread within the farm. Together, these complementary strategies contribute to reducing coinfections and morbidity and improving productivity, thereby lowering the overall impact of PRDC. PRDC, porcine respiratory disease complex.

## External biosecurity: the first line of defense

The aim of external biosecurity is preventing outside pathogens from entering pig farms. In the context of PRDC, it is essential, as pigs can encounter many of its viral and bacterial agents through the introduction of new animals, contaminated objects or vehicles, visitors and personnel, or through airborne transmission from nearby farms. Additional transmission routes may also play a role, as certain microorganisms associated with PRDC can be transmitted through semen. Strengthening the measures aimed at preventing contact with external sources of infection is crucial to minimizing PRDC outbreaks, therefore helping to better shield productivity and welfare.

### Control of animal sourcing

The breeding population due to the current nature of production systems plays a crucial role in pig herds, influencing productivity, health, and overall profitability. Replacement of breeding animals can be done internally by rearing gilts from the farm or externally, through purchase (13). Eradication of an endemic disease can get complicated when the replacement is only done internally. Purchasing breeding animals may accelerate genetic improvement, but it also carries a risk of introducing pathogens into the herd. This risk is particularly relevant because many swine pathogens spread through direct contact (14). For example, the transmission rate of *A. pleuropneumoniae* is estimated to be 10 times higher via direct contact compared to indirect routes (15). Nonetheless, if animals are purchased, they should always come from the same source farm that has a health status equal to or higher than that of the recipient farm with strict hygiene measures during transport (16).

### Quarantine and acclimation

Quarantine is a fundamental component of biosecurity in swine production, serving as the first barrier against the introduction of infectious diseases into a herd. Regarding the location of quarantine facilities, increasing distance from other pig housing areas is considered an important measure to reduce the risk of airborne transmission. Different minimum separations between the quarantine and the principal housing areas have been discussed. However, the effectiveness of these distances is highly dependent on environmental factors, production system and the specific pathogen involved. For example, some agents, such as PRRSV or *M. hyopneumoniae*, have been detected in infectious airborne particles over considerably longer distances. Experimental studies recovered infectious PRRSV from air samples collected up to 9.1 km from the source population under favourable conditions, suggesting that in certain cases additional biosecurity measures may be required beyond simple spatial separation (17).

Quarantine of incoming pigs should be followed by an acclimation programme designed to adapt them to the health status of the recipient herd. Such programmes may include vaccination against relevant endemic pathogens and, when appropriate and carefully controlled, exposure to selected resident animals, with the aim of inducing protective immunity before introduction into the herd (14, 16, 18).

It is also necessary to have a contingency plan for the event of a positive result for an unexpected disease. This contingency plan may range from extending isolation of the replacement batch until the gilts are no longer a threat, to discarding just positive individuals and extending quarantine of the others with continuous monitoring, to complete depopulation of the quarantine and monitoring of the destination farm (14).

### Semen screening

Screening of semen is a key measure in preventing the transmission of several PRDC pathogens, particularly PRRSV and PCV2 (19). To strengthen biosecurity, insemination centers should implement frequent and comprehensive testing strategies that combine complementary diagnostic methods. Serum PCR is typically the earliest and most sensitive tool for detecting viremia and bacteremia, while serology provides insight into previous exposure (20). However, semen PCR remains an essential complementary test, particularly during or following regional outbreaks or suspected exposures, since viral shedding in semen may persist even after systemic clearance. A combined approach using serum PCR, ELISA, and semen PCR ensures broad diagnostic coverage across different stages of infection and pathogen shedding (21). Additionally, oral fluid sampling represents a welfare-friendly and cost-effective strategy for herd-level monitoring, especially for PRRSV (22).

To mitigate the spread of airborne pathogens, artificial insemination centers often use air filtration systems to limit viral entry, a measure that would also be beneficial if adopted at the farm level (23). Technologies like ultraviolet C and electrostatic precipitators are among the most promising emerging approaches, potentially matching or exceeding filter efficiency in the future but still require adaptation and testing for agricultural use (24). Although this research was focused on PRRSV, these general biosecurity principles can be extrapolated to other PRDC pathogens.

### Transport, personnel and fomites control

Preventing the introduction of pathogens via vehicles, equipment and personnel is an essential component of external biosecurity. Experimental studies in pigs have shown that contaminated personnel and fomites can contribute to the indirect transmission of PRRSV and swine influenza A virus (25, 26). In addition, PCV2 and *A. pleuropneumoniae* can persist in the farm environment, highlighting the importance of cleaning and disinfection to reduce indirect transmission (27, 28). Measures such as farm-specific clothing and footwear, good hygiene practices, shower in/shower out procedures and appropriate cleaning of equipment can therefore help reduce this risk. UV light and ozone have shown potential to reduce PRRSV transmission; however, their use on pig farms remains limited (29). In addition, implementing consistent sanitation protocols for transport vehicles, including thorough washing, disinfection and drying, has been shown to reduce the risk of PRRSV transmission through contaminated livestock trailers when properly performed (30). Furthermore, the number of visitors to the farm should always be kept to a strict minimum and farm workers should avoid any contact with external pigs, with clear policies established for individuals who may have had such contact (31).

### Pest control

Wild animals like boars can have a huge role in the transmission of respiratory pathogens, in particular to pigs raised extensively. However, in intensive pig farming many diseases can be transmitted by pests, therefore making an effective pest control program essential. The continuous availability of feed and water, together with high stocking densities and favorable environmental conditions, makes livestock facilities particularly attractive to pests (32). Two main pest groups appear to have a role in the development of PRDC: birds and insects.

Wild birds are the primary natural reservoirs of the Influenza A virus. There are several reports of these avian strains being detected in pigs and they are susceptible to infection with both avian and human influenza viruses, making them important hosts for viral reassortment and the emergence of novel strains (33). Birds can transmit the virus through direct contact or through contaminated food, water or fomites (26). It is imperative to restrict their entry to the farm perimeter, for example, with bird-proof nets or screens (34).

Flies and mosquitoes have been identified as potential mechanical vectors of PRRSV, PCV2b and/or *M. hyopneumoniae* (35–38). In poultry production systems, houseflies have also been shown to acquire and mechanically carry avian influenza viruses (39, 40). Whether houseflies can similarly carry and disseminate swIAV on pig farms remains an untested hypothesis. Installing insect screens over windows and other openings and eliminating breeding sites such as standing water are recommended measures to limit mosquito entry and breeding on pig farms (41). The targeted use of approved insecticides, in accordance with label instructions and applicable regulations, can complement mosquito control measures on pig farms (41).

## Internal biosecurity: preventing the spread of diseases

Internal biosecurity, through the implementation of different farm management practices aims to limit the spread of infectious agents among animals, production stages and housing units. Since PRDC often results from interactions between multiple pathogens and environmental stressors, controlling disease transmission inside the farm is critical once pathogens are present. Internal biosecurity strategies include all-in/all-out production systems, proper cleaning and disinfection between batches, segregation of age groups, optimized ventilation, vaccination programs, and strict hygiene protocols for workers and equipment. These measures help reduce pathogen circulation, improve respiratory health, and mitigate the severity and economic impact of PRDC within pig populations.

### Workflow and weight management

Maintaining a strict pig flow is fundamental for controlling respiratory pathogens. This includes enforcing all-in/all-out management with age segregation, minimizing cross-fostering, maintaining low animal densities, avoiding the use of nurse sows and group mixing, and implementing rigorous biosecurity measures (14, 42).

Alongside these management practices, monitoring growth performance may provide additional information on the overall health status of pigs and the potential impact of respiratory disease. In this regard, high weight gain in the nursery phase is considered a good predictor of better health with stronger immune markers, lower illness scores, better antioxidant status, lower gut permeability and greater subsequent weight gain (43, 44). However, some animals may still have heavy pathogen loads, reinforcing the idea that weaning weight alone cannot capture the full picture of the development of this disease (43).

### Herd closure strategy

The herd closure technique has been demonstrated as an effective approach, particularly for the control and the elimination of PRRSV and *M. hyopneumoniae* in farms (45, 46). In this technique, no new animals are brought into the farm for an extended period while exposing the immune system of the present animals to the pathogens through vaccination or where appropriate, through controlled acclimation or exposure. The main difficulty is sustaining this negative status over time, as lapses in biosecurity can, in many cases, easily lead to reinfection (42, 47). However, the success of herd closure depends on the epidemiology of the target pathogen. For *A. pleuropneumoniae*, the persistence of clinically healthy tonsillar carriers capable of intermittent bacterial shedding may hinder elimination efforts by maintaining a reservoir of infection within the herd, even in the absence of new animal introductions (48).

In extreme cases, depopulation followed by repopulation, together with a rigorously implemented cleaning and disinfection process and subsequent intensive internal biosecurity and management practices, may be necessary to eradicate specific pathogens such as PRRSV (49). Although the economic impact of implementing an eradication program can represent a substantial initial burden, such elimination protocols may ultimately result in significant financial benefits, estimated at approximately 877,375 USD per farm per year; or about 7 USD per pig marketed in the case of *M. hyopneumoniae*, for example (50). Overall, while eradication strategies may require substantial upfront investment, they can provide long-term economic and health benefits, making them a viable option for controlling some PRDC-associated pathogens.

### Stocking density control

High stocking density may increase respiratory infection pressure by increasing the concentration and potential exposure of pigs to infectious aerosols (51). Overcrowding also increases stress and contributes to the accumulation of airborne contaminants like ammonia, thereby increasing pigs’ susceptibility to PRDC (52). For example, in pig farms where respiratory problems are caused by *M. hyopneumoniae,* a scientific review recommended a space allowance of at least 0.7 m^2^ per finishing pig housed on fully slatted floors (42). However, since respiratory disease results from interacting environmental, management and infectious factors, one universally ideal density cannot be claimed.

### Environmental control

The general conditions of the environment of a farm are essential for maintaining a healthy respiratory tract. Poor environmental conditions can damage the respiratory tract and increase susceptibility to infection. For example, experimental exposure of piglets to 80 ppm ammonia for 8 h per day over 12 consecutive days was associated with changes in the nasal mucosa consistent with ciliary dysfunction, which could compromise mucociliary clearance (53). Elevated ammonia levels have also been associated with poorer growth performance and more severe lung lesions caused by *M. hyopneumoniae* (54).

Relative humidity can affect air quality, so ventilation should be sufficient to prevent excess moisture from accumulating in pig housing. When outdoor temperatures are below the target indoor temperature, relative humidity should be maintained within the desirable range of 40–65% (55). Ventilation requirements therefore vary with seasonal conditions to balance thermal comfort and indoor air quality. During winter, reduced ventilation can limit heat loss but may promote the accumulation of humidity, dust and harmful gases, whereas during warmer periods increased air exchange and air velocity can facilitate heat dissipation and reduce heat stress (56). CO₂ and airborne dust should also be monitored as they are really important for air quality, however a universally applicable, pig-specific health threshold has not been established for either. In a commercial farrowing room, air filtration reduced inhalable and respirable dust concentrations by 25 and 48%, respectively, compared with an unfiltered control room (57).

Temperature has been demonstrated to influence pigs’ thermal comfort, feed intake and growth. The optimal temperature depends on the pigs’ age and body weight, as well as their housing conditions, with indicative values for different production stages provided by Buoio et al. (56). Together, these measures help protect respiratory function and reduce conditions that may favour PRDC.

## Diagnosis and monitoring

Due to the multifactorial and polymicrobial nature of PRDC, its diagnosis can be particularly challenging. The detection of a respiratory pathogen does not necessarily establish its involvement in the clinical disease, since several PRDC-associated microorganisms may circulate subclinically within a herd or are part of the normal respiratory microbiota. Therefore, an accurate diagnosis should integrate clinical signs, epidemiological information, pathological findings and laboratory testing to identify the pathogens most likely contributing to the respiratory condition (6, 58).

Serology represents a useful indirect approach for determining previous exposure to several PRDC-associated pathogens and can be particularly valuable at herd level. Cross-sectional serological profiles, obtained by sampling pigs from different age groups, can help determine the circulation dynamics of infectious agents within a farm, whereas paired serum samples may provide evidence of recent infection through seroconversion (8). Nevertheless, serological results should be interpreted cautiously, as the presence of antibodies indicates previous exposure rather than active infection, and antibodies induced by vaccination may interfere with the interpretation of results for some pathogens (59).

In contrast, molecular techniques such as PCR and quantitative PCR (qPCR) enable the direct detection of PRDC-associated pathogens and are widely used for both diagnostic and monitoring purposes. Nonetheless, as previously mentioned, the mere detection of a microorganism does not necessarily imply its involvement in the disease, especially in the case of opportunistic microorganisms (58). The choice of sample depends on the pathogen, the stage of infection and the purpose of testing. When necropsy is available, lung tissue or swabs collected directly from visible lung lesions are considered the gold-standard specimens for PRDC diagnosis, as lesion-targeted sampling improves pathogen recovery and facilitates the association between pathogen detection and respiratory lesions (60). Samples obtained from live animals, including oral fluids, saliva and respiratory swabs, facilitate repeated or population-level surveillance (61, 62). Moreover, the simultaneous molecular detection of multiple pathogens should be interpreted as co-detection rather than definitive evidence of active coinfection or pathogen–pathogen interaction (58).

For bacterial pathogens, culture remains especially valuable because it provides viable isolates for further characterization. Bacterial isolation from clinical cases should be followed by antimicrobial susceptibility testing, thereby supporting a more rational selection of antimicrobial treatments and contributing to antimicrobial resistance surveillance (6, 63).

Beyond the diagnosis of individual outbreaks, continuous herd-level monitoring is essential for understanding the respiratory health status of a farm and evaluating the effectiveness of preventive and control measures. On-farm monitoring can integrate clinical observations, production parameters, mortality and treatment records with periodic laboratory testing. Importantly, sampling strategies should be designed according to the pathogen and the epidemiological question being addressed rather than relying on a single diagnostic method (58). Periodic surveillance complemented with molecular characterization is particularly relevant for highly variable pathogens such as swIAV, as its continuous genetic and antigenic evolution can lead to changes in the strains circulating within pig populations (64). At a broader scale, longitudinal analysis of routine diagnostic data can reveal temporal, geographical and production-stage variations in PRDC-associated pathogen detection, thereby helping to define surveillance and diagnostic priorities (58).

Slaughterhouse monitoring provides an additional opportunity to assess respiratory health at population level. Systematic examination and scoring of lung lesions can reveal the prevalence and severity of lesions, such as cranioventral pulmonary consolidation and pleurisy, therefore providing useful longitudinal information and allowing comparisons between batches or production cycles (65). However, slaughterhouse monitoring does not capture earlier production stages in which respiratory disease may be particularly prevalent, such as the post-weaning period. In addition, slaughterhouse lesions are generally not pathogen-specific and represent the cumulative outcome of respiratory disease over the production period. Consequently, these findings should ideally be combined with farm records, clinical observations and laboratory diagnostics rather than being used alone to identify the etiological agents involved.

## Vaccination: a key tool for disease prevention

Vaccination represents one of the most effective strategies for controlling infectious diseases, serving also as an important measure to reduce the emergence of antimicrobial resistance (66). Within the framework of PRDC, vaccines hold significant promise for reducing both pathogen circulation and the reliance on antimicrobial treatments. Although vaccination plays a vital role in managing PRDC in pigs, a comprehensive and integrated approach is crucial to successfully combat the disease, encompassing biosecurity, management practices, and herd health monitoring.

### Current state and dilemmas of PRDC vaccines

#### Multivalent vaccine strategy

The coinfection dilemma of this syndrome requires a global approach that goes beyond the individual vaccines. Commercial multivalent vaccines currently provide simultaneous protection against key respiratory pathogens, particularly *M. hyopneumoniae* and PCV2, while multivalent vaccine candidates incorporating PRRSV and other respiratory pathogens are under active development (67, 68). However, although multivalent PRDC vaccines have improved practicality and coverage, combining several antigens into a single formulation often forces trade-offs in the antigen dose or adjuvant choice because antigenic competition may occur (69, 70). For instance, the immune profile that optimally protects against *M. hyopneumoniae* may not match what is required for PCV2 or PRRSV (71, 72). Furthermore, greater attention should be given to adjuvant selection, as adjuvants can help balance competing immune responses and substantially influence vaccine efficacy in multivalent vaccines (73).

#### Addressing pathogen genetic variability and evolution

Along with coinfections, PRDC remains a major challenge for swine health due to the high variability of some of its causative pathogens. Among the primary agents, swIAV and PRRSV exhibit the most rapid genetic and antigenic evolution, driven by antigenic drift and genetic reassortment in swIAV, and by mutation and recombination rates in PRRSV (74, 75). Moreover, PCV2 shows an unusually high evolutionary rate for a DNA virus, complicating vaccine design and long-term protection (76, 77). A clear example of the devastating effects that mutations can have is in the highly virulent PRRSV-1.1 mosaic strain derived from the PR40 strain that caused several outbreaks in Spain. It caused similar effects in farms as if they were encountering PRRSV for the first time, as the protection they had was no longer effective against this novel strain. This resulted in an immense impact on both swine health and production with an estimate of 18 million pigs affected in Spain (78).

Computational strategies such as Epigraph and COBRA are being used to generate broadly reactive antigens, which induce immune responses that recognize a broad range of viral strains (79, 80). Future designs may use mosaic antigens or conserved protein targets, which are approaches already explored with human influenza vaccines (81). The swine industry could also benefit from adopting a vaccination strategy similar to the one used in human influenza, where vaccine strains are reviewed and updated regularly before the epidemic season (82). Establishing both global and local surveillance programs, supported by comprehensive sequence databases, would enable faster identification of emerging variants and facilitate more proactive vaccine design (83, 84).

#### Constraints on vaccine-induced immunity

Beyond genetic variability, many different PRDC vaccines face intrinsic immunological limitations. For instance, inactivated vaccines often induce weak immunogenicity and mainly humoral responses, requiring multiple doses or strong adjuvants to enhance protection (85, 86). This general lack of robust cellular immunity leads to short-lived or unbalanced protection, as observed in many PRDC inactivated vaccines (87–89). Another major limitation of inactivated vaccines is their often-restricted heterologous protection, particularly against pathogens with substantial serotype and strain diversity, where vaccine-induced protection is often restricted to homologous or closely related serotypes or strains like *A. pleuropneumoniae*, *G. parasuis*, *S. suis*, PRRSV or swIAV (90–94).

Historically, live-attenuated vaccines have been widely used for some major swine respiratory pathogens and continue to be explored for others because they mimic natural infection and induce both good humoral and cellular immune responses (95, 96). However, their efficacy may be reduced when antigenically divergent or emerging genotypes circulate in the field, as is the case with PRRSV live-attenuated vaccines (97). In addition, these vaccines carry inherent biosafety concerns, including the potential for reversion to virulence, recombination with field strains with some having higher replication and transmission capacity; and potential spread to non-target hosts, particularly for pathogens with a broad host range (98).

The PROTAC (Proteolysis-Targeting Chimeric) approach has shown promising preclinical results in the attenuated vaccine field. This technology attenuates viruses by promoting the degradation of essential viral proteins, thereby reducing viral replication while preserving the immunogenicity. In mice and ferret models, PROTAC influenza A viruses were highly attenuated while eliciting robust humoral, mucosal, and cellular immune responses that conferred protection against both homologous and heterologous viral challenges (99). However, this technology remains at an experimental stage and has not yet been evaluated in the PRDC context, warranting further investigation.

#### Modifications of the administration route

The route of administration can greatly limit the protection of a vaccine. Most commercial PRDC vaccines are delivered parenterally, which, besides causing stress and local reactions, generally provides poor mucosal immunity (100). Since the respiratory tract is the primary entry site for PRDC pathogens, local IgA-mediated immunity is essential to block initial infection (98). Intranasal vaccines have been on the rise in these recent years, with promising protective efficacy demonstrated against several PRDC pathogens, including *M. hyopneumoniae*, *G. parasuis*, PRRSV and swIAV (66, 101–103). Alternative delivery methods, such as aerosolized or oral-feed vaccines are also being explored. However, their main challenges lie in ensuring sufficient antigen uptake and maintaining vaccine stability (104, 105). However, follow-up research is still needed to determine whether upper nasal delivery, deep lung delivery, or a combination of both provides the best protection (104). Devices and platforms also need to be optimized to ensure that vaccines reach the desired respiratory portion.

#### Optimizing vaccination through combined and tailored approaches

Given the multifactorial nature of PRDC and the limitations of current commercial vaccines, alternative and tailored vaccination approaches are increasingly being explored to enhance immune coverage, improve cross-protection, and better target respiratory immunity in the context of PRDC.

##### Prime-boost technique

The development of different strategies of vaccination is being explored, with an emphasis on the prime-boost technique. Its aim is to stimulate different complementary components of the immune system by priming with one vaccine platform and boosting with another, so that the strength of each one is harnessed (106). For example, the prime vaccine may elicit strong mucosal and T-cell immunity, followed by a booster to enhance systemic antibody responses. In the PRDC context, heterologous prime-boost vaccination has shown promising results against PRRSV by enhancing the breadth of the immune response and improving protection against heterologous strains (107). However, there is a need for expanding this technique to other pathogens in addition to optimizing the combinations through more field tests that examine the best timing for each vaccine and the best antigen combination.

##### Autogenous vaccines

Another tailored option adopted by many farms is the use of autogenous vaccines. Due to frequently limited cross-protection of many vaccines, these vaccines are designed to match the strains circulating within individual herds and are relatively common in the prevention and control of PRDC-associated pathogens (108–110). The Regulation (EU) 2019/6 establishes autogenous vaccines as an exceptional tool, intended primarily for situations in which no suitable authorized veterinary vaccine exists for the microorganisms involved in a particular farm. Their production is labour-intensive and time-consuming, and their use is restricted to the originating herd, thereby limiting their broader applicability (111).

#### Limited large-scale field validation

Furthermore, a major limitation in the evaluation of veterinary vaccines, therefore including those targeting PRDC-associated pathogens, is the limited availability of large-scale field studies assessing their performance under commercial conditions (112). Although controlled challenge studies often appear promising, vaccines must be tested under real-world conditions to generate comprehensive information on their efficacy. Thus, while these studies provide valuable evidence of vaccine efficacy, their findings should be complemented by large-scale field studies to determine whether the observed protection translates to commercial conditions (112). Multiple factors including maternal antibody interference, the developing piglet immune system, interactions between different vaccines and coinfections, can all influence vaccination outcomes, and should be considered in these trials (43). Large-scale field trials can help refine vaccination protocols by identifying vaccination timing, booster requirements, and the influence of maternal immunity under real-world conditions (113). Moreover, long-term post-licensure evaluations are imperative to better assess vaccine effectiveness and long-term safety under commercial production conditions.

### Next-generation vaccine developments for PRDC

#### Nanoparticle-based vaccines

New vaccine platforms are continuously being explored, with a big push for nanoparticle-based vaccine platforms. One promising example is the development of vesicle-based vaccines against *A. pleuropneumoniae*. These vesicles possess intrinsic adjuvant and antigenic properties. Since they originate from the bacterial outer membrane, they naturally carry a wide repertoire of outer membrane proteins, toxins, porins, and adhesins, providing a multivalent antigen composition that closely mimics natural infection. In mice, *A. pleuropneumoniae*-derived vesicles have elicited stronger immune responses than commercial vaccines (114). However, in pigs, although high levels of IgG were achieved, this was not translated into improved lung protection (115).

Virus-like particles (VLPs) represent a novel protein nanoparticle vaccine platform. VLPs are non-infectious, self-assembling protein structures that closely mimic the organization and morphology of native viruses while lacking viral genetic material, thereby providing a favorable safety profile. Their virus-like architecture and highly repetitive antigen display can efficiently stimulate both humoral and cellular immune responses, supporting their development as vaccine platforms (116). This vaccine platform is being actively investigated in pigs for its potential to induce broader and longer-lasting protective immunity (117, 118).

Virus-like particles (VLPs) have emerged as a promising vaccine platform for several PRDC pathogens. PCV2 represents the most successful example, as its capsid protein self-assembles into VLPs that closely resemble native virions, enabling the development of highly immunogenic commercial vaccines with scalable production (117–119). VLP-based vaccines have been investigated as safer alternatives to modified live vaccines (MLVs), with chimeric and baculovirus-derived VLPs inducing encouraging humoral and cellular immune responses against both PRRSV-2 and PRRSV-1 (120, 121). Although these findings do not demonstrate protection in pigs, they provide preliminary proof of concept supporting the subsequent evaluation of PRRSV-specific VLP vaccines in the natural host. Similarly, VLPs expressing hemagglutinin (HA) and/or neuraminidase (NA) have shown promising results against swIAV by eliciting broad immune responses and providing protection following viral challenge, supporting their potential as cross-protective influenza vaccines (122, 123).

It should be noted that for bacterial members of PRDC, the VLP concept does not directly apply since they lack capsid structures. Nevertheless, advances in subunit and multiepitope vaccines in pigs are improving the immunogenicity and protective efficacy against bacterial pathogens, as demonstrated in *A. pleuropneumoniae, M. hyopneumoniae* and *G. parasuis* (103, 124, 125). In addition, chimeric proteins containing potentially immunogenic domains from *M. hyopneumoniae* antigens induced humoral and cytokine responses in a preclinical murine model, although their immunogenicity and protective efficacy remain to be confirmed in pigs (126).

#### Vectored vaccines

Viral vectored vaccines can provide a durable, broad and protective vaccine platform that can be rapidly updated by changing their antigenic composition to fit emerging variants (127, 128). When a new viral variant has emerged, researchers do not need to redesign the entire vaccine platform. Instead, they replace the antigen-encoding gene while keeping the same vector backbone.

In PRDC-relevant pathogens, adenovirus, vaccinia (MVA) and PRV vectors have shown immunogenicity in mice or pigs, and in some cases, protection in pigs (107, 129, 130). Other viral vectors, such as Newcastle disease virus, are also being investigated for the development vaccines against swIAV (131). Computational tools such as Epigraph have been widely used to rapidly generate and update vaccine antigens. Recently, this approach was applied to develop a successful swIAV viral-vectored vaccine in pigs (132). Although these studies have focused on swIAV, the same design strategy could be explored for other PRDC-associated viruses, such as PRRSV or PCV2, which also exhibit high genetic variability.

#### Nucleic acid-based vaccines

From a broader vaccine-development perspective, DNA vaccines represent a rapidly updatable and non-infectious platform. Although the integration of plasmid DNA into the host genome remains a theoretical safety concern, experimental evidence indicates that this risk is extremely low. Modern vector designs, including optimized plasmids and minicircle DNA vectors lacking unnecessary bacterial backbone sequences, may further improve their safety and expression profiles (133, 134). In pigs, a lipid nanoparticle-encapsulated DNA vaccine induced protective immunity against both swine- and human-origin H1N1 influenza viruses, illustrating the potential of optimized DNA delivery systems for controlling porcine respiratory infections (135).

Messenger RNA (mRNA) vaccines offer a particularly favorable safety profile, as they are non-infectious and entirely circumvent the possibility of genome integration associated with DNA-based platforms. These vaccines elicit robust immune responses by directing host cells to synthesize pathogen-derived antigens, and their sequences can be readily modified to address emerging variants (136). Recent progress has been made in developing mRNA vaccines against PRRSV, with several studies demonstrating robust immunogenicity and protection outcomes in mice and piglets (137–139). An mRNA vaccine candidate against a secondary PRDC pathogen, *M. hyorhinis*, demonstrated higher IgG titers and improved daily weight gain in immunized pigs, although it did not confer sufficient protection against infection (140). Overall, these findings indicate that mRNA vaccine technology represents a promising and rapidly advancing approach within the context of PRDC; however, the available evidence remains limited, and further studies are required to confirm its protective efficacy under large-scale field conditions.

## RNA-based therapeutic and diagnostic approaches

An interesting targeted, although still experimental, therapeutic approach against PRDC-associated viruses is the use of small interfering RNAs (siRNAs) and microRNAs (miRNAs). These small RNAs can guide the RNA-induced silencing complex (RISC) to specific RNA targets, resulting in their degradation or translational repression and thereby reducing the expression of specific proteins. Such approaches could potentially be used against PRDC-associated viruses by targeting viral RNA or host factors required for viral replication, while miRNAs are also being investigated as potential diagnostic biomarkers (141). Several studies have explored these possibilities in the context of PRDC-associated viruses, including swine influenza virus and PRRSV (142–144). Similar targeted strategies are also being investigated against bacterial pathogens using antisense oligonucleotides (145). Nevertheless, these approaches remain largely at the experimental stage.

## Natural alternatives to antibiotics

Historically, antimicrobials have played a central role in mitigating bacterial components of PRDC and limiting associated economic losses in intensive pig production systems (146). However, the widespread and recurrent use of antibiotics, often at group level during potential high-risk moments such as weaning, mixing or transportation, has contributed to the selection and dissemination of antimicrobial-resistant bacteria within pig populations (146, 147). Several bacteria associated with PRDC exhibit high levels of antibiotic resistance and harbor antimicrobial resistance genes, many of which are located on mobile genetic elements. A notable example is *S. suis*, posing a potential risk to human health due to the increasing evidence of horizontal transfer of antimicrobial resistance genes to human streptococci (148). Increasing regulatory restrictions on prophylactic and metaphylactic antimicrobial use; as exemplified by the European Union, together with growing public health concerns, have intensified the need for effective non-antibiotic strategies (111, 149). In this context, natural alternatives to antibiotics are being actively explored as complementary tools to support respiratory health, enhance host resilience and reduce antimicrobial dependence in PRDC management. Among the most promising natural alternatives, probiotics, essential oils, organic acids and bacteriophages have recently attracted significant public and scientific interest. To better understand their potential role in the swine respiratory field, Figure 2 summarizes the main mechanisms of action of these various alternatives to antibiotics.

**Figure 2:**
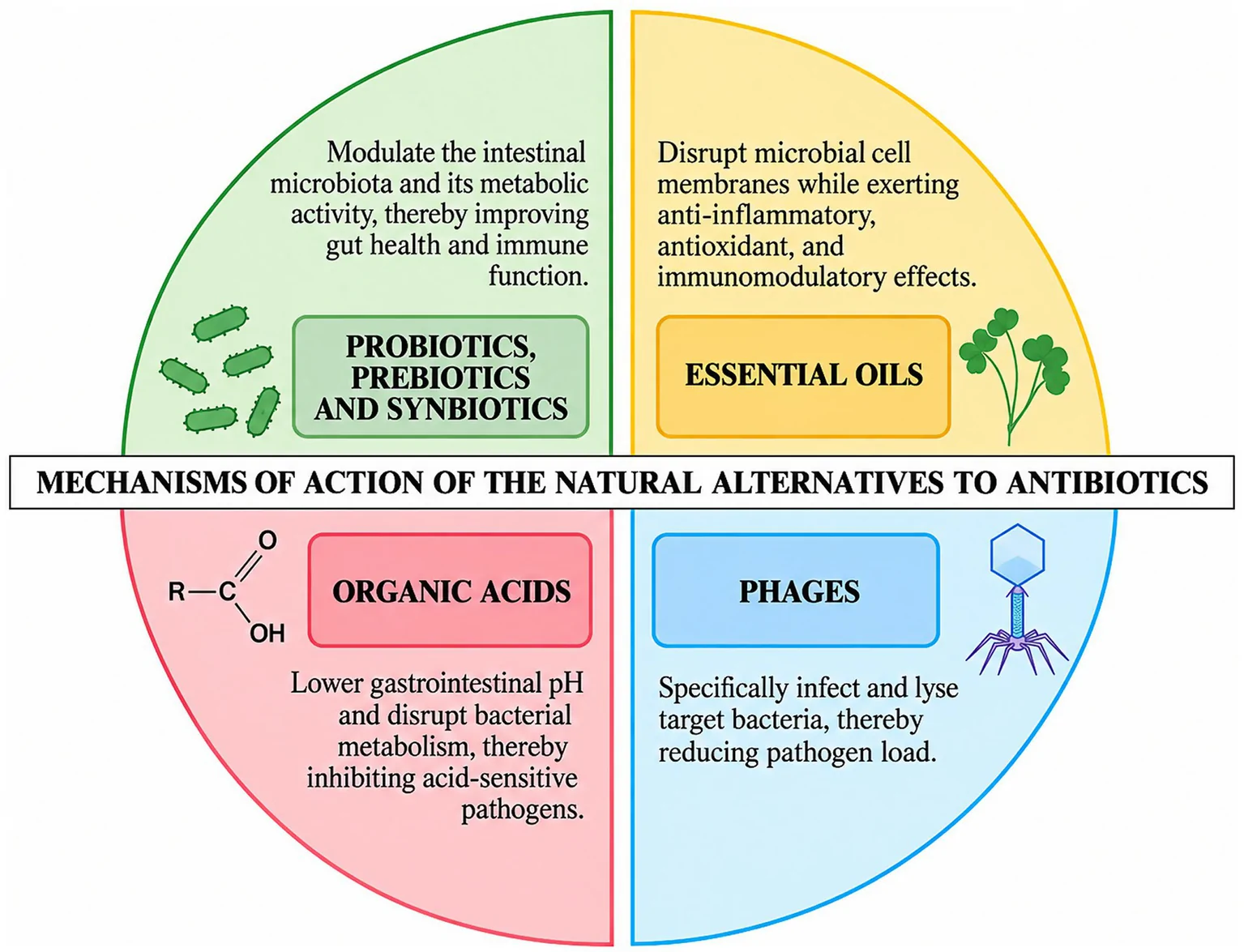
Principal mechanisms of action of natural alternatives to antibiotics investigated against PRDC-associated pathogens. Each alternative is presented according to its predominant mechanism of action; however, additional biological effects not depicted in the figure may also occur. PRDC, porcine respiratory disease complex.

Many of these natural alternatives act systemically or through the gut-lung axis, thereby indirectly shaping respiratory immune responses (150, 151). The gut-lung axis allows for changes in intestinal immunity to influence pulmonary defenses and vice versa, for example, by enhancing macrophage activity, regulating cytokine production or improving vaccine responses (152). As evidence for the double pathway mechanism, in mice Influenza A has been able to modify the gut microbiome composition by promoting the migration of Th17 cells from the lungs to the intestine, even though it did not colonize the intestinal tract (153). In another study also performed in mice, they detected that Influenza A can induce early upregulation of intestinal lymphoid cells like ILC1s and ILC2s (154). Although much of the evidence of this connection is derived primarily from humans or mice, some articles also support this connection between gut and lung in pigs. For instance, in the case of *M. hyopneumoniae*, early-life gut bacteria, particularly members of the family *Ruminococcaceae*, were linked with reduced lung lesions in pigs (155). To further illustrate this connection, pigs with a diverse and balanced gastrointestinal microbiome have been associated with milder disease symptoms, reduced viral replication and improved growth performance following PRRSV and PCV2 infections (156, 157). Collectively, these indirect actions can modulate the susceptibility to respiratory infections and vaccine efficacy, indicating their preventive and supportive roles in mitigating PRDC.

Importantly, reducing antimicrobial dependence in PRDC management should not rely exclusively on the development of alternative products. Prudent antimicrobial use also requires treatment decisions to be supported by clinical and laboratory diagnosis whenever feasible, with antimicrobial susceptibility testing contributing to the selection of appropriate agents, particularly in recurrent cases or following treatment failure. At herd level, optimizing the indication and timing of group treatments may further reduce unnecessary antimicrobial exposure.

### Probiotics, prebiotics and synbiotics

Probiotics are live microorganisms that confer health benefits when consumed or applied in the correct amounts (158). Other related compounds are prebiotics and synbiotics. Prebiotics are a substrate that is specifically used by the host microorganisms whereas Synbiotics are a mixture of live microorganisms and substrates. The substrate in the Synbiotic mixture can be designed to be utilized by the co-administered microorganism or by the host microbiota (159). They all act mainly through indirect mechanisms originating in the gut, in which they can reshape the intestinal microbiota, enhance epithelial barrier integrity, and modulate both local and systemic immune responses (160).

Probiotics represent an emerging, though still relatively underexplored area within the PRDC context. A summary of studies investigating probiotics in this context is provided in Table S1. Recent advances have led to the development of genetically engineered and recombinant probiotics designed to specifically target respiratory pathogens (161, 162). In the context of PRDC, a notable advance has been the expression of PRRSV antigens in probiotic bacteria. For instance, research done in 2025 reported *L. plantarum* strains expressing fragments of an anti-PRRSV monoclonal antibody. Oral administration of these recombinant strains helped neutralize the virus at mucosal surfaces and promoted a protective immune response following PRRSV challenge (163). To further illustrate the ongoing research in this area, a 2024 study developed a *B. subtilis* spore-based oral vaccine displaying GP5 glycoproteins from PRRSV-1 and -2 where vaccinated pigs exhibited significant IgG and IgA responses, along with elevated cytokine levels, indicating a robust immune activation (105).

PRDC-specific research for prebiotics and synbiotics is extremely limited since almost the entirety of the studies in pigs are focused on growth, gut health or systemic biomarkers rather than direct respiratory endpoints. However, one study evaluated the effects of a type of prebiotic, the mannan oligosaccharides (MOS) in pigs experimentally infected with PRRSV. They reported improved feed efficiency and higher antibody titres during the chronic phase of infection, although no reduction in viremia was observed (164). In addition, another study by the same research group found that dietary MOS supplementation altered the response of porcine alveolar macrophages to ex vivo LPS stimulation, reducing TNF-α secretion and increasing IL-10 secretion (165). These findings suggest that these compounds represent a promising avenue for further research as potential alternatives to antibiotics.

### Essential oils and organic acids

Essential oils are among the most extensively investigated groups of phytobiotics in swine production. Dietary supplementation with selected essential oils may influence gut microbial composition, oxidative stress, inflammatory pathways, and immune function, although their effects vary considerably depending on their composition, dosage, formulation, and bioavailability (166). A summary of studies investigating essential oils in the context of PRDC is provided in Table S2. Essential oils represent a promising yet largely unexplored strategy for PRDC control, and considerable research is still needed to clarify their efficacy, safety, and practical application in swine production.

Organic acids are compounds that contain carbon and one or more acidic functional groups and are widely used particularly for their antimicrobial and growth-promoting effects (167). As demonstrated for essential oils, research on organic acids as potential tools against PRDC pathogens remains limited and largely focused on intestinal health. There is a broader body of literature that describes how organic acids influence respiratory health in humans and other animals (168–170). However, studies addressing respiratory effects in pigs are scarce, although their results are promising. For instance, fogging a mixture primarily containing formic and propionic acids was shown to alleviate several symptoms of enzootic pneumonia (171). Regarding PRRSV, organic acid-based disinfectants have demonstrated antiviral activity against the virus *in vitro* (29), while feed additives containing organic acids were evaluated as part of a comprehensive next-generation biosecurity program under commercial conditions, where they were used to mitigate the impact of PRRSV-contaminated feed (172).

Research on nano-encapsulated essential oils is emerging in the prophylactic field as it can improve the delivery and dispersion by improving the stability, bioavailability and guaranteeing a controlled and exact release, but their application in pigs remains largely unexplored. In this context, an *in vitro* study against human respiratory pathogens suggests that essential-oil nanoemulsions may possess broad antimicrobial activity (173). However, these results cannot be directly extrapolated to PRDC-associated bacteria, and its efficacy against relevant PRDC isolates remains to be established.

### Bacteriophages

Bacteriophages (or phages) constitute promising alternatives to antibiotics because their generally narrow host range enables the selective targeting of bacterial pathogens and may cause fewer broad alterations to the commensal microbiota than conventional antibiotics. Several lytic bacteriophages with antibacterial properties against PRDC agents have been described, for example; the vB_BbrP_BB8 bacteriophage can cause the lysis of *B. bronchiseptica*; or the vB_PmuP_PHB01 with *P. multocida* (174, 175). Furthermore, an *in vitro* study demonstrated the antibiofilm activity of phage SS2 against *S. suis*; however, its therapeutic efficacy in pigs remains to be established (176). However, research on PRDC-associated bacteria has largely focused on isolated temperate bacteriophages, prophages, or prophage regions identified solely through in silico prediction (177–180). Although these elements are valuable for understanding phage diversity and identifying potentially useful lytic enzymes, they do not necessarily provide phages suitable for therapy. Further work should therefore prioritize the isolation and characterization of strictly lytic phages against these bacteria. Despite the limited evidence currently available, phage-based approaches remain a promising area of research for controlling PRDC bacteria.

### Current limitations in the development of natural alternatives to antibiotics

Overall, the available evidence indicates that many studies investigating these approaches rely heavily on *in vitro* or non-porcine *in vivo* models, most commonly mice. Significant interspecies differences in drug metabolism, pharmacokinetics, and other physiological characteristics can render a drug that is effective and safe in mice, ineffective or even toxic in pigs (181). Robust *in vivo* evidence in pigs, particularly for respiratory outcomes, is still heavily limited and there is a lack of standardization such as in the outcome measures, the population studied or environmental and management factors. For example, in the case of the influenza virus, little is researched in pigs, as most information available on alternatives to antibiotics is centred on the human or avian influenza virus.

Furthermore, these alternatives typically exert most of their biological effects through indirect mechanisms. When these compounds are administered orally their bioavailability may be reduced by gastrointestinal absorption, metabolism, and first-pass hepatic processing, making the amount of active compound reaching distant target tissues difficult to predict. Consequently, many of their reported respiratory benefits are thought to arise from indirect mechanisms, such as modulation of the gut–lung axis, rather than from the direct accumulation at the respiratory epithelium (150, 182). Encapsulation of these compounds has improved their stability and bioavailability, although further research is needed to evaluate their efficacy in pigs (183).

In addition, translation to field efficacy remains constrained by dose-dependent intake or tolerance effects like reduced feed intake due to palatability problems or adverse effects when overconsumed. A dose-related safety concern arises particularly with prebiotics, which mainly consist of non-digestible carbohydrates (dietary fibre). High inclusion levels of prebiotics, if not accompanied by appropriate diet reformulation, may dilute dietary energy and nutrient density, ultimately leading to reduced feed efficiency (184). Moreover, inconsistent outcomes of these alternatives may arise from differences in formulation processes, including lyophilization, dosage form, and storage conditions, as well as from the lack of standardization of these practices in academic research (185–187). In addition, the chemical composition of essential oils can be influenced by seasonal and geographical factors, which can alter their biological activity and, consequently, their therapeutic efficacy (188, 189). Furthermore, the predominantly single-pathogen design of these studies limits the extrapolation of their findings to PRDC coinfections under commercial conditions.

When these alternatives are used alone, without complementary interventions such as vaccination, local airway pathogen loads may remain unaffected. Exploring alternative delivery approaches, such as inhalation or aerosolization formulations could provide promising new directions for future research as they could act directly at the site of infection and could potentially limit the pathogen load.

## Future perspectives

Future perspectives in the control and prevention of PRDC are shaped by the ongoing challenges associated with its multifactorial nature, high coinfection rates and the limitations of current strategies. While considerable progress has been made, important gaps remain in achieving consistent, sustainable and integrated disease control. Addressing these challenges will require continued research efforts and the exploration of innovative approaches to improve existing tools and develop new interventions.

One of these key challenges is the optimization of vaccination strategies. While vaccines against major PRDC pathogens have significantly reduced disease impact, their effectiveness is often compromised by antigenic variability, coinfections and suboptimal immune responses in the respiratory mucosa (74, 75, 100, 190). Future vaccine development should move beyond single-pathogen protection and aim to account for polymicrobial contexts. This includes refining multivalent formulations, improving antigen selection to minimize immune interference, as well as exploring novel adjuvants and delivery routes, to enhance local immunity at the site of pathogen entry. Nonetheless, large-scale field trials in pigs should also remain a primary objective of PRDC vaccine research.

To further improve treatment of pigs, greater efforts are needed to improve the delivery and standardization of non-antibiotic alternatives, such as probiotics, essential oils, organic acids, and bacteriophages. While research in these areas remains limited, many of these strategies show promise. However, their field efficacy remains inconsistent, likely due to variations in formulations, dosing regimens, delivery routes, and study designs. Given that much of the current evidence comes from *in vitro* studies or mouse models, future research should prioritize studies in pigs and develop standardized protocols to evaluate the efficacy of these compounds against respiratory disease. Still, it is important to acknowledge that these alternatives are designed to support and enhance existing management and biosecurity practices, by modulating the susceptibility to respiratory infections and vaccine efficacy.

Finally, future control of PRDC will increasingly rely on integrated and adaptive management strategies, with particular emphasis on robust biosecurity. Integrating molecular diagnostics with continuous health surveillance, production performance indicators, and environmental monitoring may facilitate earlier and more informed interventions. This integrated approach could also enable more targeted use of vaccines and therapeutics, contributing to a shift from reactive to proactive disease management. Such approaches will be essential to reduce antimicrobial dependence while maintaining animal health and productivity in increasingly restrictive regulatory frameworks.

## Conclusion

The evidence reviewed highlights that PRDC should be approached as a herd-level disease complex rather than as the sum of individual respiratory infections. Its multifactorial and polymicrobial nature means that the detection or control of individual pathogens does not necessarily translate into effective control of respiratory disease at the population level. Consequently, biosecurity, herd management, appropriate diagnosis and monitoring, and vaccination remain the foundation of PRDC control and should be considered complementary rather than independent measures.

At the same time, the strength and applicability of the available evidence vary considerably among control strategies. Established measures are supported by extensive experience in swine production, whereas many next-generation vaccines and alternatives to antibiotics remain at earlier stages of development or have predominantly been evaluated under experimental conditions. These emerging approaches therefore represent promising additions to, rather than replacements for, established PRDC control measures.

Overall, the evidence indicates that successful PRDC control depends less on any single intervention than on selecting and combining measures according to the pathogens involved, herd health status, management conditions and epidemiological context. Thus, progress in PRDC control will likely depend not only on developing new technologies, but also on using available and emerging tools more strategically. This integrated and context-specific approach may ultimately provide the most sustainable route to reducing respiratory disease and antimicrobial dependence while maintaining pig health and productivity.
