# Supplementary material for: Control and prevention strategies of porcine respiratory disease complex: current evidence and future directions
Source: Front Vet Sci. 2026 Oct 27;13:1992010. doi: 10.3389/fvets.2026.1992010 (PMC13750139; doi:10.3389/fvets.2026.1992010)
Supplement: Supplementary file 1 [file Data_Sheet_1.PDF]

Table 1. Potential effective natural probiotics against PRDC pathogens.

Pathogens	Probiotics	Type of study	Probiotic dose in in vivo studies	Duration of administration of the probiotics	Main outcome measures	References
M. hyopneumoniae	*Saccharomyces cerevisiae*	Porcine experimental challenge.	• Disintegrated/fragmented *S. cerevisiae*: 0.25 g/animal/day.<br>• Live *S. cerevisiae*: 0.50 g/animal/day.	Initially from birth (day 0) to day 21 in feed. Then from day 21, oral forced intake for 7 consecutive days before the *M. hyopneumoniae* infection challenge.	Pigs that received *S. cerevisiae* showed improved clinical scores, fewer lung lesions and better weight gain. The fragmented probiotic preparation showed the greatest reduction in lung lesions.	160
S. suis	*Lactococcus lactis* subsp. *lactis* L2	In vitro.	Not applicable.	Not applicable.	Bacteriocin-like inhibitory substances of the probiotic showed bactericidal inhibition against *S. suis*.	161
B. bronchiseptica	*Bacillus subtilis, Lactobacillus acidophilus* and *Saccharomyces cerevisiae*	Non-porcine in vivo (mice).	Diet supplemented with 2% (w/w) fermented Maesil (FM). The FM had been fermented with 1 × 10^7 CFU/mL of each probiotic organism during preparation.	Mice were fed the FM diet for 4 weeks before challenge with supplementation continued in the infection	The mice who had the probiotics administered produced more macrophages and T cells; and showed a higher titre of anti-*B. bronchiseptica* antibodies. They also showed a stronger cytokine response after the challenge.	162

Table 1. Potential effective natural probiotics against PRDC pathogens.

				experiments.		
**PCV2**	*Lactobacillus acidophilus*	Non-porcine in vivo (mice).	• Gavage: 10^8, 10^6 or 10^4 CFU/mouse.<br>• Enema**:** 10^8, 5×10^7 or 2.5×10^7 CFU/mouse.<br>Before the PCV2 challenge the groups received 10^8 CFU/mouse/day.	Probiotic was administered on days 1-5 and 11-15. The viral challenge was performed on day 10.	The probiotic showed anti-PCV2 effects by lowering viral gene copies and lowering IL-1β expression. The enema administration promoted greater *L. acidophilus* colonization, although most antiviral and anti-inflammatory outcomes did not differ significantly between administration routes.	163
**PCV2**	*Lactobacillus acidophilus*	In vitro and in silico.	Not applicable.	Not applicable.	Some metabolites of *L. acidophilus* like glutamine can have anti-PCV2 activity.	164
**PRRSV-2**	*Lactobacillus casei* subsp. *rhamnosus*	Porcine experimental challenge.	Initial intranasal dose: 6×10^9 CFU/pig in 2 mL PBS; thereafter 6×10^9 CFU/pig/day in drinking water.	Started at 25 days of age and administered daily until the end of the trial at 74 days.<br>PRRSV vaccination was performed at day 30; PRRSV	When the probiotic was administrated in combination with a PRRSV vaccine, higher weight gain post-infection was shown.	165

Table 1. Potential effective natural probiotics against PRDC pathogens.

				challenge at day 56.		
**PRRSV-2 + *Salmonella enterica* serovar Choleraesuis**	*Bacillus subtilis*	Porcine experimental challenge.	3.67×10^5 CFU/g feed.	Started 3 weeks before challenge and continued through the challenge period.	The probiotic decreased the PRRSV viral load and enhanced the innate immune response. It also lowered *Salmonella* colonization in the lungs and improved lung pathology scores.	166
**PRRSV-2**	*Enterococcus faecalis*-HH69, *Enterococcus faecium*-HH32-1 and *Limosilactobacillus reuteri*- HH09	In vitro.	Not applicable.	Not applicable.	The probiotic showed strong antiviral properties as it reduced PRRSV replication in vitro. They also produced lower levels of pro-inflammatory cytokines, including IL-6, IL-8, TNF-α, and TGF-β1. *L. reuteri* increased the expression of the antiviral gene ISG15, whereas *E. faecium* enhanced the expression of another antiviral gene, OAS1.	167

Table 1. Potential effective natural probiotics against PRDC pathogens.

**PRRSV-2 + PRV**	*Gordonia alkanivorans,*	• In vitro: PRRSV<br>• Porcine field study: PRV	0.67, 2.0 and 6.0 kg/ton feed.	28 days.	The action of the probiotic strongly enhanced the phagocytic activity of porcine alveolar macrophages (PAMs) against PRRSV in vitro. It also improved immune responses in the PRRSV-related assays by modulating: IFN-α, IFN-γ, TNF-α, IL-1β, IL-2 and IL-10. In the field study, pigs previously vaccinated against PRV showed an increased antibody response against PRV following the probiotic supplementation.	168
**swIAV (H1N2)**	*Bacillus subtilis*-597	Porcine experimental challenge.	2.6×10^6 CFU/g feed	Started 14 days before swIAV challenge and continued during the 7-day infection period.	Pigs who were administered the probiotic showed a reduction of lung lesions and an attenuated increase of C-reactive protein when compared to pigs who did not receive the probiotic. A decreased expression of interferon-stimulated genes was detected in the probiotic group.	169

Table 1. Potential effective natural probiotics against PRDC pathogens.

**swIAV (H1N1) + PRRSV-2 + PRV**	*Bacillus subtilis*-NS12	• PRRSV: in vitro and porcine experimental challenge<br>• swIAV (H1N1): in vitro<br>• PRV: in vitro and non-porcine in vivo (mice)	• PRRSV pigs: CFU dose not specified<br>• PRV mice: 1 × 10^8 CFU	• PRRSV: 3 intranasal administrations during 3.5 days: days 1 and 3, followed by PRRSV challenge 24 h after the second administration, and an additional NS12 administration 12 h post-challenge.<br>• PRV: 5 consecutive days of intranasal NS12 administration;	• PRRSV/swIAV in vitro: the probiotic, *B. subitilis* NS12 strong antiviral activity, with inhibition of viral genes, protein expression and suppression of infectious viral titres.<br>• PRRSV in vivo: NS12 protected pigs by lowering serum and lung viral loads and reducing pulmonary lesions.<br>• PRV in vitro: NS12 showed strong antiviral activity. The probiotic produced metabolites such as surfactin and piceatannol, which altered viral envelope properties and blocked viral entry.<br>• PRV in vivo: NS12 was protective in mice, with improved survival and reduced viral loads and tissue damage.	170

Table 1. Potential effective natural probiotics against PRDC pathogens.

				PRV challenge occurred on day 2 of the administration period.		

Abbreviations: CFU, colony-forming units; FM, fermented basil; IFN, interferon; IL, interleukin; ISG15, interferon-stimulated gene 15; OAS1, 2′-5′-oligoadenylate synthetase 1; PAMs, porcine alveolar macrophages; PBS, phosphate-buffered saline; PCV2, porcine circovirus type 2; PRRSV, porcine reproductive and respiratory syndrome virus; PRV, pseudorabies virus; swIAV, swine influenza A virus; TGF-β1, transforming growth factor beta 1; TNF-α, tumor necrosis factor alpha.
