# Supplementary material for: Control and prevention strategies of porcine respiratory disease complex: current evidence and future directions
Source: Front Vet Sci. 2026 Oct 27;13:1992010. doi: 10.3389/fvets.2026.1992010 (PMC13750139; doi:10.3389/fvets.2026.1992010)
Supplement: Supplementary file 2 [file Data_Sheet_2.PDF]

Table 2. Potential effective essential oils against PRDC pathogens.

**Pathogen**	**Extracted plants**	**Major components studied**	**Type of study**	**Concentrations and activity**	**Main outcome measures**	**References**
**PRRSV-2**	*Eucalyptus* spp (the species are not identified).	Not specified.	In vitro.	0.0156, 0.0312 and 0.0625% EO; 0.0625% was the maximum safe concentration used for the detailed virucidal experiment.	Strong virucidal activity when added with PRRSV before cell infection, suggesting it has the potential of being a preventive and disinfectant tool against PRRSV-2.	177
**PRRSV** (the type is not specified)	*Cinnamomum iners* Reinw. and *Cinnamomum burmanii* Blume.	Cinnamaldehyde derived benzimidazoles.	In vitro.	Selected non-cytotoxic concentrations: approx. 4 nL/mL *C. iners* EO and 2 nL/mL *C. burmannii* EO.	*Cinnamomum iners* benzimidazole showed no inhibition of PRRSV replication while the *Cinnamomum burmannii* benzimidazole appeared to enhance the infection in the pre-infection assay and showed 20% inhibition in the post-infection assay.	178
A. pleuropneumoniae, A. suis, B. bronchiseptica, G. parasuis, P.	*Abies balsamea*, *Cinnamomum verum*, *Coriandrum sativum*, *Ledum groenlandicum*,	Carvacrol, cinnamaldehyde and thymol.	In vitro.	• Serial EO concentrations 1.25–0.01% v/v for MIC/MBC.<br>• Biofilms were treated	At concentrations effective against the bacteria, these essential oils did not impair pig tracheal epithelial cell viability. Thyme and winter savory significantly reduced biofilm viability for *S. suis* and *A. pleuropneumoniae.* Strong synergistic	179

Table 2. Potential effective essential oils against PRDC pathogens.

multocida* and *S. suis	*Mentha piperita*, *Origanum majorana*, *Salvia officinalis*, *Satureja montana* and *Thymus vulgaris*.			at 1× and 2× MIC.	interactions between the bacteriocin nisin, and thyme or winter savory essential oils were observed against *S. suis*.	
A. pleuropneumoniae	*Thymus* spp (the species are not identified).	Thymol.	In vitro and non-porcine in vivo (mice).	• In vitro MIC: 31.25 µg/mL<br>• Mice: 20 mg/kg thymol, subcutaneous.	High bactericidal activity from the thymol was detected in the in vitro assay. In addition, biofilm and virulence genes were altered or downregulated in the in vitro assay. In a mouse model, a protection against lethal infection and clinical signs was found.	180
A. pleuropneumoniae	*Cinnamomum zeylanicum*, *Coriandrum sativum*, *Eucalyptus citriodora*, *Mentha piperita*, *Mentha spicata*, *Laurus nobilis*,	Alcohols, aldehydes, monoterpenes and phenylpropanoids.	In vitro.	• MIC testing: 0.009–5 mg/mL.<br>• Biofilm disruption: 0.125–4× MIC.<br>• Biofilm prevention: 0.125, 0.25 and 0.5× MIC.	Cinnamon and coriander oils had the most antibacterial activity. Cinnamon, coriander, peppermint, eucalyptus and laurel oils disrupted biofilm formation by up to 80%. Subinhibitory concentrations of cinnamon and laurel oils significantly reduced biofilm formation.	181

Table 2. Potential effective essential oils against PRDC pathogens.

	Origanum majorana and *Thymus vulgaris*.					
A. pleuropneumoniae* and *S. suis	The species are not explicitly identified.	Galangal oil, monoterpenes, oregano oil, phenylpropanoids (eugenol) and methoxybenzene.	In vitro.	Varied by compound and bacterial species.	Eugenol showed the strongest activity against *A. pleuropneumoniae* (MIC of 0.49 mg/mL). Oregano oil also showed broad antibacterial activity against *A. pleuropneumoniae* and *S. suis*.	182

Abbreviations: EO, essential oil; MBC, minimum bactericidal concentration; MIC, minimum inhibitory concentration; PRDC, porcine respiratory disease complex; PRRSV, porcine reproductive and respiratory syndrome virus; v/v, volume/volume.
